# A Human Full-Skin Culture System for Interventional Studies

**Published:** 2009-01-09

**Authors:** Lars Steinstraesser, Andrea Rittig, Kai Gevers, Michael Sorkin, Tobias Hirsch, Marco Kesting, Michael Sand, Sammy Al-Benna, Stefan Langer, Hans-Ulrich Steinau, Frank Jacobsen

**Affiliations:** ^a^ Department for Plastic Surgery, Burn Center, BG University Hospital Bergmannsheil, Ruhr University Bochum, Bochum, Germany; ^b^ Department of Oral and Maxillofacial Surgery, Klinikum Rechts der Isar, TU Munich, Germany

## Abstract

**Objective:** Novel approaches to bridge the gap between clinical studies and experimental basic research of skin physiology are urgently needed. The aim of this study was to develop an effective surrogate model in which ex vivo full-thickness organ culture experiments may be performed. **Methods**: Human full skin from patients was placed into a stainless steel chamber and cultured at an air-liquid interphase for 4 weeks. Samples were evaluated every week by HE-staining and immunohistochemical characterization. Epidermal gene transfer kinetics was performed as an interventional study. **Results**: This ex vivo chamber model maintained the physiologic and histologic properties of the skin explants for 4 weeks. This indicated the model's acceptable ex vivo physiologic validity. No epidermolysis was observed, and both basal lamina and blood vessels were detected within all tissue samples. Transgene expression was demonstrated to be time dependent. **Conclusion**: This model chamber presents a convenient, easy-to-use, and robust model in which ex vivo full-thickness organ culture experiments may be performed.

Further research into the cellular and molecular functions of the skin may lead to improvements in our understanding and management of wounds, but transitional research from the laboratory to the clinic is not always straightforward.

Different therapies that affect wound repair have been proposed over the last few decades. To gain deeper insights into the biology and pathophysiology of wound healing, as well as to develop new therapies such as gene therapy, it is paramount to employ convenient, easy-to-use, and robust experimental models that are versatile and allow translatability.

Studies investigating skin disorders in humans are limited owing to ethical concerns, leading to dependence on in vitro and experimental animal models to investigate novel therapies and biologic and pathophysiologic pathways.^1^ Many models have been developed,^2^ but in vitro and in vivo models may show poor consistency with clinical situations.^3^

In most cases, the wounds being investigated are artificially and, importantly, acutely induced in the normal, healthy animal that serves as the model. However, most animals exhibit cell biology, histology, immunology, and biochemistry distinct from humans, with different healing processes, unique complications, and nonequivalent tissue structures (eg, the contraction of the subcutaneous muscle *panniculus carnosus*, which is absent in humans, and not by reepithelialization). In addition, animal behavior, therapeutic needs, and skin healing responses (eg, inflammation, healing mechanisms, and kinetics) also differ from those of humans. The use of animal models to study wound healing under various stimuli and applied therapeutic methods is often an attempt to most expediently duplicate conditions closest to the human patient. Interspecies differences in anatomy and physiology, differences in cause and course between natural human disease and artificially induced nonhuman pathology, and stress experienced by animals in laboratories invariably alter research results.

Few in vitro or nonmammalian surrogate experiments accurately or adequately duplicate, or recapitulate, the full physiology of the human model for wound healing purposes.^3^ Many research efforts seek new innovative approaches to in vitro modeling of in vivo complexity without clear progress or improved relevance.

In vitro human models help investigate skin physiology and wound healing in a more equivalent fashion. The major advantage of in vitro models is the opportunity to analyze environmental changes, substrate and dose-response interactions, and cutaneous immune responses in a standard fashion.^2^,^4^

Wound closure is usually simulated in vitro by simply creating defects in cell monolayers, and the repopulation of the defect by adjacent cells through the combined action of migration and proliferation is then monitored.^5^ However, these models do not reflect the complex process of wound healing because cell cultures of primary cells or cell lines neither have a three-dimensional structure nor show any immune response and systemic interaction. These systems lack the multidimensional aspects to evaluate wound healing.

As three-dimensional systems are more representative for normal wound physiology than 2-dimensional systems,^6^ several approaches to employ three-dimensional cell cultures have been reported, including prefabricated coculture systems, three-dimensional gels with incorporated fibroblasts, and keratinocytes cultured on the apical side of the gel.^7^,^8^ However, cells cultured in three-dimensional matrices typically composed of type I collagen or fibrin significantly alter their phenotype.^9^ Extensive phenotypic changes of contractile fibroblasts cultured in three-dimensional matrices are observed owing to altered mechanical tensions within the systems. Moreover, three-dimensional models are labor intensive and require expert skills to employ these systems.

A major obstacle in gene therapy studies is the extrapolation from in vitro to the complex interactions of multiple different immunocompetent cells in the wounded skin.^3^,^10^ To understand wound healing and its potential therapies in a greater detail, it is better to investigate full-skin biology and pathophysiology. Therefore, ex vivo organ culture may be promising because it maintains the cutaneous structure and allows for the evaluation of morphology, physiology, and biochemical activity.^3^

In contrast to skin equivalents, all cellular elements and their interactions are included in human full-skin culture explants. Therefore, this approach offers improved translation between the investigative laboratory and the clinical setting.

Wound healing studies in skin explants have been performed previously,^3^,^11^ and in most cases, epithelialization was analyzed.^12^ In addition, the change in tensile strength can be monitored in the experimental setting.^13^ Standardized burn wounds may also be inflicted in skin explants and assessed for epithelialization by histomorphometry.^14^ As a result, this model provides the opportunity to carry out morphologic studies of the connective tissue in wound healing.^15^

As it is possible to generate wounds in skin explants, it is also possible to pursue their potential treatment under in vitro conditions. To discriminate between topical, intradermal, or subdermal treatment, the used model should have discrete superior and inferior compartments, which are strictly separated by the cultured tissue. Therefore, skin explants should be cultured at the air-liquid interface, which induces physiologic maturation of keratinocytes to cornified cells and ensures the preservation of skin barrier function.^16^,^17^

The aim of this study was to develop an effective surrogate chamber model for the ex vivo investigation of the human skin. In this study, human skin explants were cultivated at an air-liquid interphase by using a newly designed stainless steel chamber, and to confirm this model's feasibility for the study of human skin biology and pathophysiology and novel therapeutic approaches, dermal wounding and gene transfer were performed and monitored over time. The delivery of therapeutic agents by medium, intradermal or topical application was assessed and transgene expression, transgene concentrations, and local effects were investigated.

## MATERIALS AND METHODS

### Tissue

Skin explants were obtained from adult healthy patients (age range = 22–54 years) undergoing breast reduction or abdominoplasty surgery. The study was approved by the local ethics committee, and all of the patients gave written informed consent. Immediately postexcision, the human skin was washed in antiseptic (Octenisept, Schuelke-Mayr, Norderstedt, Germany) 3 times for 10 seconds.

### Organ culture

Human skin tissue was washed several times in phosphate buffered saline (PBS) and subcutaneous fat was excised. Next, the tissue was sliced into triangular pieces of 2.5 × 2.5 cm. These pieces were transferred to the base of a stainless steel chamber, placing the epithelial site upward. The upper part of the chamber was bolt down until the tissue was fixed, and during fixation, the skin explants were stretched to prevent contraction. Samples were cultured at the air-liquid interphase by using a 6-well plate filled with 5 mL of culture medium; DMEM (Gibco, 21969-035, Paisley, England), containing 10% FBS (Hyclone, Logan, Utah), 1% penicillin/streptomycin, and amphotericin B (25 μ g/mL, PAA, Pasching, Austria). The tissue was incubated at 37^°^C in a humidified atmosphere containing 5% CO_2_, and the medium was changed twice a week.

### Epidermal wounding

Skin explants from healthy patients were prepared and cultured for at least 4 weeks. For epidermal wounding, the epidermis was scored by using a 6-mm punching knife and a scalpel. Fifty microliters of a 0.2% dispase solution was dropped onto the scored epidermis. After 3 hours at 37^°^C, the epidermis within the 6-mm area was completely removed (Fig 6*a*). Adenoviral vectors (10^8^ IU) were topically applied with or without the removal of the epidermis. Two days later, tissue biopsy specimens were harvested. Following this, 10-μm tissue sections were taken and X-gal staining was performed.

### Production and purification of recombinant adenovirus

For gene transfer, replication-deficient human Δ E1 type 5 adenoviruses with inserted CMV-promoter–driven *Escherichia coli* β-galactosidase^18^ was used. The virus was propagated in 911 cells, purified by 2 sequential CsCl_2_ gradients, and dialyzed against 20 mM of Tris-HCl, pH 8.0. The titer was determined by Adeno-X rapid titer kit (Becton Dickinson Bioscience, K1653-1, Heidelberg, Germany). Virus stocks were stored at –80^°^C in 10% glycerol.

### Quantification of reporter gene expression

Skin explants were intradermally injected with 10^8^ adenoviral vectors containing the transgene for *E coli* β-galactosidase. After 2, 3, 5, 7, and 14 days, the skin samples were weighed and homogenized in PBS by using a rotor stator homogenizer (Polytron PT3100, Kinematika, Luzern, Switzerland). Homogenates were centrifuged at 14,000 rpm at 4^°^C for 2 minutes (Eppendorf centrifuge 5402, Hamburg, Germany). The supernatant was measured by Galacto-Light-Plus (Tropix, Lincoln, Neb) in a microplate luminometer (Berthold, Orion, Pforzheim, Germany). The amount of reporter gene product was adjusted to total protein by using a BCA protein assay reagent kit (Pierce, Rockford, Ill) in a microplate reader (Bio-Tek, EL_X_-808, Winooski, Vt).

### Immunohistochemistry

Biopsy specimens were taken, fixed in 4% neutral buffered formalin, embedded in paraffin, and finally 4-μm sections were prepared. For histological assessment, a standard HE-staining of these sections was performed.

After heat fixation, deparaffinization antigen unmasking, and blocking, slides were incubated with the primary antibody of the corresponding antigen: antihuman Ki-67 antibody (Dako, Hamburg, Germany) at a dilution of 1:75, mouse antihuman caspase-3 antibody (Acris, Hiddenhausen, Germany) at a dilution of 1:150, and the rabbit antihuman laminin antibody (Sigma, St Louis, Mo) at a dilution of 1:30. The slides were incubated overnight at 4^°^C, rinsed with PBS several times, and incubated with a corresponding biotinylated secondary antibody for 30 minutes at room temperature (RT). After the washing procedure, sections were incubated in streptavidin Alexa Fluor488 conjugate for 30 minutes at RT. The slides were rinsed again and DAPI counterstaining was performed. Finally, the slides were covered with fluorescent mounting medium (Dako, Hamburg, Germany). Pictures were taken by using an Axioskop 2 plus microscope (Zeiss, Jena, Germany) connected to an AxioCam HRC camera (Zeiss, Jena, Germany) at 50- to 400-fold magnification. High-powerfield counting was performed at 200-fold magnification. Each value represents the mean of 10 high-powerfield countings.

### X-gal staining

After 4 weeks of culture of the human skin explants (*n* = 4), a 6-mm punch biopsy (Stiefel, Offenbach a.M., Germany) was performed to create an epidermal defect in 2 of these explants. The prepared area was covered with 50 μL of 0.2% dispase solution and incubated for at least 3 hours at 37^°^C. Following this, dispase was stopped by rinsing the wound with FCS and PBS and the epidermis was peeled off. Adenoviral vectors (10^8^ IU containing LacZ transgene) were topically applied to each skin explant (*n* = 4) and the tissue was again cultured for 2 days. Next, the tissue was harvested, embedded into O.C.T. compound (CellPath, Newtown Powys, England), and conserved in liquid nitrogen. Frozen sections (10 μm) were obtained and fixed in neutral buffered 0.05% glutardialdehyde for 5 minutes at RT. Sections were washed with PBS several times and the X-gal (Roth, Karlsruhe, Germany) staining solution was added followed by a 6-hour incubation at 37^°^C. Successful staining was controlled by microscopy and counterstained by using nuclear fast red (Sigma, Taufkirchen, Germany).

### Statistical analysis

All assays were performed in triplicate. Data were analyzed by using analysis of variance and independent samples *t* test (SPSS, Chicago, Ill). A *p* value of less than .05 was considered significant.

## RESULTS

### Human skin chamber model

A constant transformation of the epidermis was observed over the time course of experiment. The epidermal layer decreased in both dimension and structural organization, and the cell amount decreased to 37.5% at day 14 compared with the initial measurement (88 cells/HPF ± 7) and reached plateau from day 21 (55 cells/HPF ± 5) until day 28 (53 cells/HPF ± 6).

**Figure 1:**
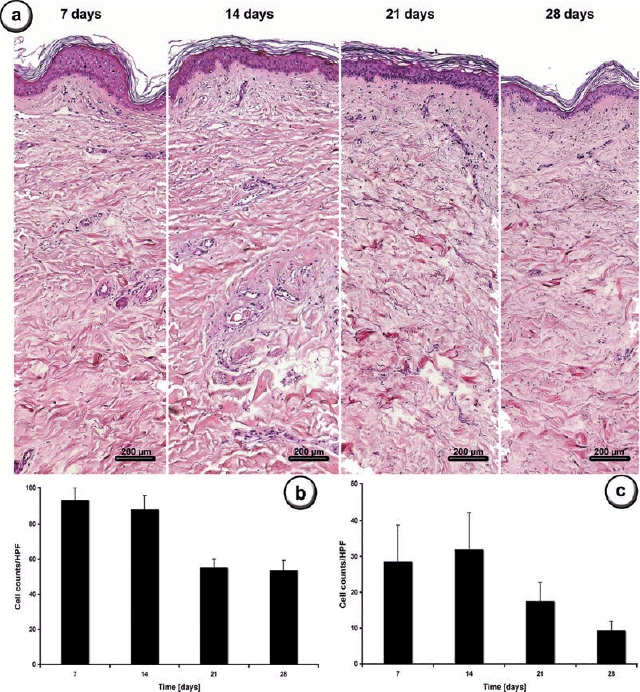
*Histological analysis*. HE-stained cross-sections of skin tissue samples (a). Modification of the dermal structure was seen in the 4-week observation period. Spaces seen after 7 days were filled up with collagen matrix until day 28. Epidermal layer decreased continuously during the observation period. High-power field analysis of the sections demonstrating the amount of cells within the epidermis (b) and the dermis (c). No tissue necrosis was observed over the time course of 28 days. Images from 5-μm formalin fixed tissue sections at a 50-fold magnification.

The epidermis of skin explants also showed changes in its morphologic structure. Until day 7, the cells of the basal layer were strictly organized and aligned in a parallel fashion. From day 14 onward, these cells started to lose their organizational structure and spindle-shaped cells were observed. Starting at day 21, postoperative gaps were observed within the junction of the epidermis and the dermis (Fig 1).

Minimal changes could be observed in the dermal cells between day 7 (29 cells/HPF ± 10) and day 14 (32 cells/HPF ± 10). The collagen fibers were structured casually within the tissue sections of day 7, and there was a loss of connectivity at day 14. The missing crosslinks and fiber structures were reorganized following day 14, whereas the cell numbers continuously decreased to 18 cells/HPF (±5) at day 21 and 9 cells/HPF (±3) at day 28 (Fig 1).

Higher magnification of the collagen matrix and cultured dermis showed that although the collagen fibers were much thinner in the very young cultured skin, the collagen clearly remained organized into fibers within the whole period of culture.

### Immunostaining

**Figure 2:**
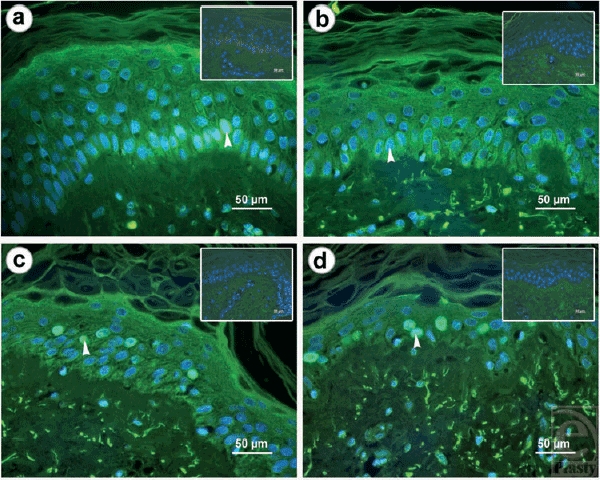
*Cell proliferation*. Immunohistochemical staining of the proliferation marker Ki-67 is shown. A monoclonal mouse antibody against human Ki-67 was used to detect proliferating cells (green). A DAPI counter stain was also performed to determine the nuclei of the cells. Images were obtained at the corresponding wavelength and merged together to show the localization of Ki-67 signal within the nucleus of proliferating cells (white arrow head). At day 7, Ki-67 was strictly concentrated in the basal layer (a). At days 14, 21, and 28, the signal became more widely distributed across the whole epidermis. This distribution was associated with an epidermal loss of structure during the 4-week follow-up (day 28) (d). A control staining without using the anti Ki-67 antibody is shown in the upper right margin of each image.

Skin samples were cultured for a 4-week follow-up. Every week, 1 sample was removed, formalin fixed, paraffin embedded, and sliced into 4-μm sections for further analysis. A change in the number of Ki-67-positive keratinocytes was not observed, whereas a change was observed in the organization and epidermal localization. At the beginning of skin culturing, a high amount of Ki-67-positive cells were observed within the basal layer of the epidermis. During maturation of the tissue specimen, Ki-67-positive cells were more and more distributed in a randomized fashion within the complete epidermis (Fig 2).

**Figure 3:**
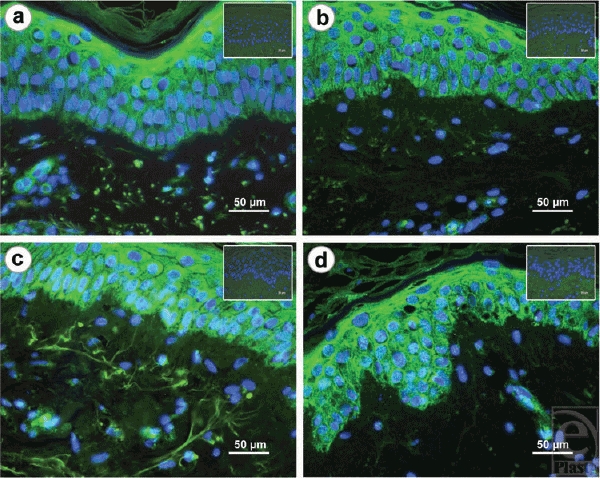
*Detection of apoptosis*. To determine cellular apoptosis in the tissue sections, immunohistochemical staining of caspase-3 was performed. Apoptosis was detected within the *stratum granulosum* (a). Fourteen days after beginning of the experiment, first apoptotic cells were visible in the *stratum basale* (b). During the following weeks (21 days (c) and 28 days (d)), an increased staining of the complete epidermal layer was observed. A control staining without using the anti caspase-3 antibody is shown in the upper right margin of each image.

To detect cellular apoptosis, tissue sections were labeled for caspase-3. Positive stained cell were exclusively found within the upper epidermal layer of the *stratum granulosum* at day 7. Caspase-3-positive labeled cells were also observed within the *stratum spinosum* at day 14 and in the basal layer after 3 weeks of culturing. At day 28, the entire epidermis was stained for apoptosis (Fig 3).

**Figure 4:**
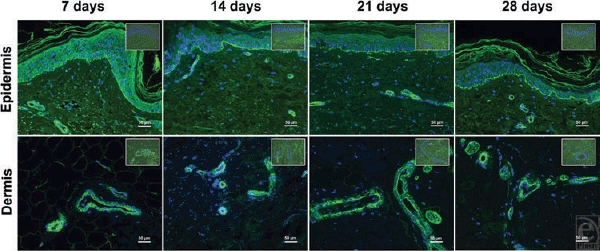
*Laminin staining*. The immunohistochemical staining of laminin (green) determined the existence of a basal lamina as an indication for the structural and functional stability of the epidermis. Furthermore, laminin staining was associated with remaining blood vessel structures. The upper layer represents the staining of epidermis, whereas the lower line demonstrates a representative staining of the dermis. Four-week follow-up (7, 14, 21, and 28 days) is shown from left to right. Control staining without using the antilaminin antibody is shown in the upper right margin of the corresponding image.

The alteration in the relationship between the constant number of Ki-67-positive cells and the increased level of caspase-3-positive stained cells on the one hand and the growing allocation of proliferative cells within the epidermis on the other hand indicated the proceeding of tissue degradation. However, laminin labeling confirmed the presence of a basal lamina between the epidermis and the dermis within all sections analyzed (Fig 4). In addition, the number of detected blood vessels was not different in comparison with the 4-week follow-up; indicating the concomitance of vital endothelial cells. These findings demonstrated the structural and functional behaviors of the organ components within the cultured tissue section.

### Viral vector–based gene transfer

**Figure 5:**
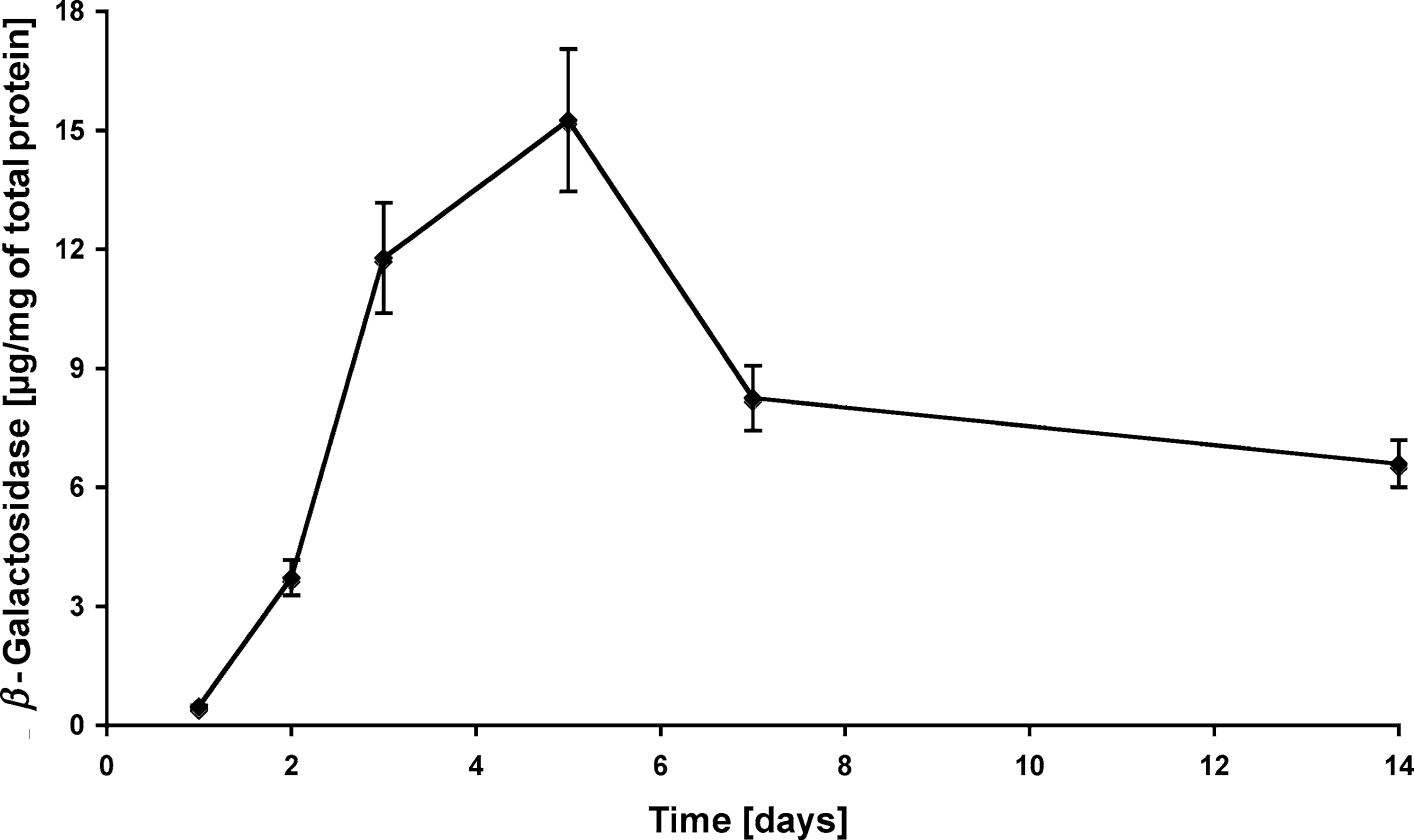
*Adenoviral gene delivery*. Skin specimens were cultured as described and transduced intradermally by using 10^8^ IU (infective units) of adenoviral vectors containing the *E coli* LacZ transgene. After 1, 2, 3, 5, 7, and 14 days, tissue samples were homogenized and the transgene product was measured quantitatively. The concentration of *β*-galactosidase was normalized to the total protein amount of the sample measured by BCA-assay. Values are displayed as mean ± SEM.

Cutaneous gene delivery was performed by the intradermal injection of adenoviral vector (10^8^ IU). The time course of transgene expression showed a significant effect for time dependency, confirming to previously performed gene delivery studies to rat skin using the same adenoviral vectors.^19^,^20^ An increased transgene expression was observed immediately after the vector application that peaked at day 5 (15.3 μg/mg of total protein). However, the transgene expression did not decrease to the background level as seen in in vivo studies, but it remained at the lower expression level (6.6 μg/mg) until the experiment ended at day 14 (Fig 5).

### Epidermal barrier function

The skin defect was further used to investigate the barrier function of the epidermis. Adenovirus cannot penetrate the *stratum corneum* of healthy skin to transduce any cells in lower skin levels. The aim of this part of the study was to detect any leakage of the epidermal layer by adding adenoviral vectors on top of the cultured skin. The adenovirus penetrates only the skin if the barrier function of the epidermal layer is damaged. Therefore, skin tissue explants remained in ex vivo culture for more than 4 weeks. A defined part of the epidermal layer was enzymatically removed and topical adenoviral gene delivery was performed with or without partial depleted epidermis.

**Figure 6:**
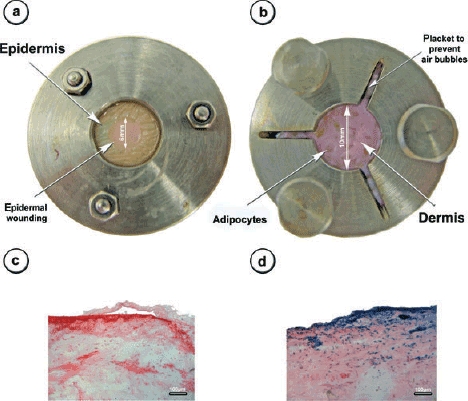
*Skin culture model*. The images of the upper row show the stainless steel chamber model from the epidermal (a) and the dermal (b) side of view. The macroscopic appearance of the epidermal wounding procedure is also displayed (a). After 4 weeks of culture and partial depletion of the epidermal layer, skin specimens have been topically transduced by using 10^8^ IU of LacZ-adenoviral vectors. Samples were taken further 2 days later and transgene expression was visualized by using X-gal staining. Only tissue specimen with removed epidermis showed transgene expression after the topical application of the virus (d, blue-stained cells), indicating intact skin barrier only for tissue without epidermolysis (c). Images are at 100-fold magnification; scale bar displays 100 μm.

If the epidermis remained intact on the specimens, no transgene expression was observed (Fig 6*c*). Without the barrier function of the epidermis, the topical application of adenoviral vectors containing the transgene for *E coli* β-galactosidase resulted in positively transduced cells within the epidermal and dermal layer tissue sections, as determined after X-gal staining (Fig 6*d*).

These findings indicate that it was possible to inflict a wound of defined dimension. In addition, the tissue remained vital as the adenoviral vector actively transduced cutaneous cells and a high-level gene expression was exhibited.

## DISCUSSION

The organ culture may be the only technique to investigate the complexity of skin tissue physiology ex vivo and may therefore bridge the gap between the investigative laboratory and the clinical setting. Moll et al^21^ foresaw that the organ skin culture model could investigate wound healing process because complex biologic aspects could be observed without any systemic influence. Reliable and robust models are needed to bear the potential of an entire physiologic epithelial environment. In many prior skin organ culture models, explants were sliced into small pieces to prevent excessive dermal contraction and cultured without any tension.^21^–^23^ This approach minimized potential opportunities and made interventional studies impossible because the pieces under investigation were too small to allow compartmentalization.

This study has established a human full-skin model that allows work with the human skin under defined in vitro conditions. A stainless steel chamber was used to apply tension to the cultured full skin to prevent dermal contraction due to the retraction forces of the elastic fibers and physiologically support skin tissue at an air-liquid interface.

The physiologic turnover of healthy human epidermis is approximately 14 days. This fact seems to be the most important reason why it is still difficult to culture human skin tissue for more than 2 weeks without losing its barrier function. The protocol of Lu et al^24^ for a long-term human scalp skin culture enabled them to investigate the physiology of hair follicles for more than 2 weeks. Krugluger et al^22^ reported the possibility of more than 8 weeks of human skin culture, but they used these skin pieces as a well-structured matrix to study the behavior of cultured cells derived from isolated hair follicles. The percentage between proliferating and apoptotic cells needs to be constant to facilitate physiologic rebuilding activity. Therefore, the presence of proliferation and apoptosis was determined by immunohistochemical staining of Ki-67 and caspase-3 in this study. During the 4-week follow-up, the distribution of Ki-67-positive cells dislocated from a structured fashion within the basal layer to an unstructured shape within the complete epidermis. Vice versa, the number of apoptotic cells increased during the same time frame with enhanced levels of distribution from the *stratum granulosum* to the whole epidermal layer. Ki-67-positive keratinocytes were frequently found at the basement layer of the epidermis within the healthy skin.^25^ In contrast, the caspase-3 level correlates with the degree of keratinocyte differentiation and showed highest level at the corneal level of the epidermis.^26^ These findings indicate a continuous degradation of the epidermis within the observation period of 28 days, which leads into a complete detachment of the epidermal layer if the tissue is cultured further.

Maintenance of skin tissue in the organ culture is dependent on intact physiologic dermal function. Dermal regulation of epidermal physiology and how this is brought about at the molecular level are not yet fully understood; this model may shed light on a number of different interfering pathways involved.

This model generates 2 different compartments separated by the clamped skin that allows substrate application to the epidermal or the dermal side of the tissue. In the context of the current restrictions on the use of animals, this model might also provide the opportunity to reduce or finally substitute in vivo animal trials.

Skin equivalents have a higher permeability by 1 or 2 orders of magnitude, making them more stable for culturing than human skin explants.^17^ Without tissue contraction, the permeability of the explants was constant; this allowed optimal delivery of nutrients to the tissue in this study and may explain the absence of epidermolysis in the cultured tissue.

After establishing this full-skin model, we applied adenoviral vectors for gene transfer to investigate the feasibility of this model for interventional studies. After gene transfer, we could show that the highest expression level at day 5 was followed by a decrease during 14 days of observation. In vivo gene delivery to rat skin was previously performed by using the same adenoviral vector system. Transgene expression was detected only within the first week after gene delivery, whereas no expression was measured after 14 days.^19^,^20^ So far, no mechanism could be described in detail that may be responsible for the decrease of transgene expression while the gene itself could be detected for weeks.^27^ This study did not attempt to define the reason for this time dependence, but an intracellular feedback regulation of the increasing transgene expression level is a possible candidate.^28^,^29^

It should be emphasized that in previous studies, skin explants were cultured in physiologic concentrations of calcium.^23^ for no longer than 2 weeks^15^,^30^ to prevent detachment of the epidermis and degradation of the connective tissue, respectively. In our study, culture medium was used that contained 1.8 μM of CaCl_2_, which could not prevent tissue transformation and partial degradation. But no epidermolysis was observed within the tissue culturing time of more than 4 weeks.

Topical gene delivery was feasible only when the epithelial layer was removed and no transgene expression could be observed if the adenoviral vector was added topically to the cultured human skin. This was observed after the tissue remained in culture for 28 days, indicating the physiologic intact barrier function of the present epidermal layer.

The protein expression of positive transduced tissue results indicated that skin explants were vital and metabolically active during the observation period of 28 days. These data demonstrate that the in vitro full-skin organ culture system is feasible to closely monitor cell metabolism over time.

After 4 weeks of skin culture, no necrotic areas were seen within the tissue sections and adenoviral-mediated transgene delivery was still successful. This again indicated that the physiologic barrier function of the epidermis remained intact. Further applications of this model are needed to demonstrate the physiologic integrity of this model in greater detail and to show how close this model comes to natural conditions. Using this model, we could inflict defined wounds into the cultured skin explants. The epidermis was completely removed within a predefined circle and the surrounding tissue remained unimpaired. Thus, this model may be used for wound healing studies focusing on the analysis of reepithelialization and wound closure. Keratinocytes undergo several kinds of behavioral changes, including proliferation, migration, and differentiation,^31^ during reepithelialization. However, further studies are clearly needed to demonstrate whether keratinocytes can be activated in this model.

In conclusion, the model described provides 2 compartments that could be treated or analyzed separately for more than 4 weeks and the chamber presents a convenient, easy-to-use, and robust model in which ex vivo full-thickness organ culture experiments may be performed.

We thank Dr Michael Wehmoeller from Cranio Construct Bochum for the production of the chambers. We also thank the Department of Pathology of the Ruhr-University Bochum for expert technical assistance.
